# Copolymer Self-Assembly Triggered by *Cis*–*Trans* Polyproline Isomerization

**DOI:** 10.1021/acsmacrolett.6c00340

**Published:** 2026-09-03

**Authors:** Ernesto Tinajero-Díaz, Antxon Martínez de Ilarduya

**Affiliations:** Departament d́Enginyeria Química, Universitat Politècnica de Catalunya , ETSEIB, Diagonal 647, 08028, Barcelona, Spain

## Abstract

Polyproline exists in two forms, the all-*cis* right-handed polyproline helix (type I) and the all-*trans* left-handed polyproline helix (type II). While the former is favored in common organic solvents, the latter is favored in aqueous solutions. Taking advantage of the hydrophilicity of type II polyproline, we prepared peptide-based nanostructures from a series of block copolymers of ε-caprolactone and l-proline. Copolymers were synthesized via *N*-carboxyanhydride polymerization in DMF using amino-ended polycaprolactone as initiator and covering a broad range of polyproline compositions. By simple stirring in water, the *cis–trans* isomerization of polyproline triggered their solubilization, leading to the spontaneous self-assembly of the copolymers into colloidally stable nanoparticles. Optical rotation measurements demonstrated the mutarotation of prolyl residues and circular dichroism spectroscopy shed light on the preferential type II secondary structure of polyproline that stabilizes nanostructures. Nanodevices encapsulated doxorubicin hydrochloride, highlighting their potential as drug delivery systems.

## Graphical abstract

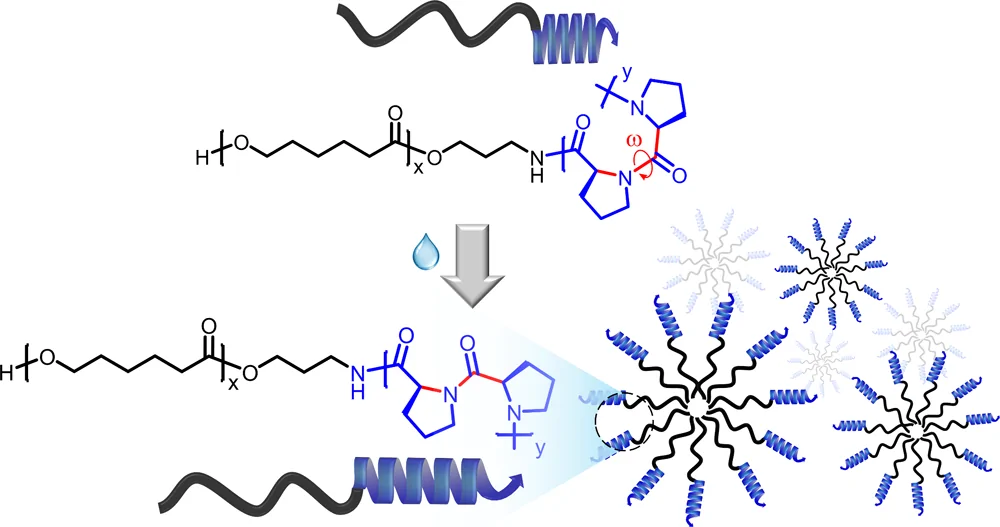

Molecular self-assembly is the spontaneous association of molecules under equilibrium conditions into stable, structurally well-defined aggregates joined by non-covalent bonds.^1^ In nature, biomolecules including peptides and proteins, interact to self-organize into complex structures that dictate biological functions.^2^ In this regard, molecular self-assembly is an excellent approach to design novel supramolecular architectures. Peptide-assembled nanocarriers, for example, hold promise for biomedical science and nanotechnology.^3^ They can be prepared via the self-assembly of amphiphilic copolymers (ACP), in which the constituent hydrophilic and hydrophobic blocks can be purely synthetic (such as polyethers or polyesters) or bio-based (such as carbohydrates or polypeptides), or a combination of both.^4^ Hence, polymeric materials used for preparing nanoparticles must be at least biocompatible and, ideally, biodegradable. A special case involves amphiphilic–hybrid copolymers, a class of molecules made of synthetic and bio-based blocks. They have attracted considerable attention because combine the advantages of biodegradability and biocompatibility.^5^

Synthetic polypeptides, i.e., poly­(α-amino acids), are a unique class of macromolecules able to mimic the properties of natural proteins and have great interest because of their potential applications in biomedicine.^6^ The ring-opening polymerization (ROP) of α-amino acid *N*-carboxyanhydrides (NCAs) offers an easy access to artificial peptides with predictable molecular weights and narrow dispersities.^7^ Furthermore, synthetic polypeptides stand out by their capacity to adopt α-helix and β-sheets secondary structures as proteins do, offering an extra tool to modulate various properties in advance materials.^8^

Among the 20 canonical amino acids, proline is unique because its side chain is cycled to the backbone, thus giving proline an exceptional rigidity and a considerably restricted conformational space.^9^ For example, the most prevalent proline-rich protein in nature is collagen, which is the most abundant protein in vertebrates and the main component of the extracellular matrix.^10^ Consequently, designing nanocarriers incorporating poly-l-proline (PLP) can mimic the behavior of natural collagen, significantly improving biocompatibility.^11,12^ Recently, important advances in NCA polymerization have been achieved, and PLP can be readily prepared by ROP of l-proline NCA.^13–16^

PLP exists as either all-*cis* right-handed type I (PLPI) helices, where all peptide bonds are in the *cis* configuration with backbone dihedral angles of (Φ, ψ, ω) = (−75°, +160°, 0°) or as all-*trans* left-handed type II (PLPII) helices with (Φ, ψ, ω) = (−75°, +145°, 180°).^17^ PLP was first synthesized in 1954 by Katchalski and coworkers,^18,19^ and their seminal work disclosed the mutarotation of PLP in solution by optical rotation. They found that such change in optical rotation was strongly influenced by both the history of the sample, i.e., the synthesis route, and the manner in which the solution is prepared. Further studies have demonstrated that PLPI is favored in common organic solvents like aliphatic alcohols, while PLPII is favored in aqueous solutions.^17^

The unique conformational properties of PLP have inspired others to drive precise molecular self-assembly. In 2022, for instance, Bonduelle et al.^20^ demonstrated that homopolyproline exhibited temperature-driven aggregation in water with hysteresis, which was tied to a change in its secondary structure. Their investigation paved the way for designing thermoresponsive, protein-like nanovesicles.^21^ PLP, on the other hand, has been copolymerized with other polypeptides.^13,22–25^ In 2024, Bonduelle, Heise and coworkers independently demonstrated how PLPII secondary structures stabilized polypeptide nanoparticles, mimicking PLPII stabilization in natural proteins.^25,26^ Lu et al. used PLP for decorating the enzyme uricase obtaining a therapeutic nanomaterial with minimal loss of enzyme activity.^27^ Similarly, copolymers of PLP and synthetic polymers have been reported,^14,28–32^ but as far as we know, the genuinely *cis–trans* isomerization of PLP has never been exploited as a peptidically-intrinsically mechanism for producing stable, hybrid nanostructures in solution.^33^

In this Letter, we report the synthesis of hybrid copolymers of poly­(ε-caprolactone) (PCL) and PLP and thoroughly characterized them. Following synthesis and isolation, and by simple dissolution in water, the copolymers were able to self-assemble triggered by the *cis–trans* isomerization of prolyl residues that promoted their solubilization to form colloidally stable micellar nanoparticles (Scheme 1).

**Scheme 1:**
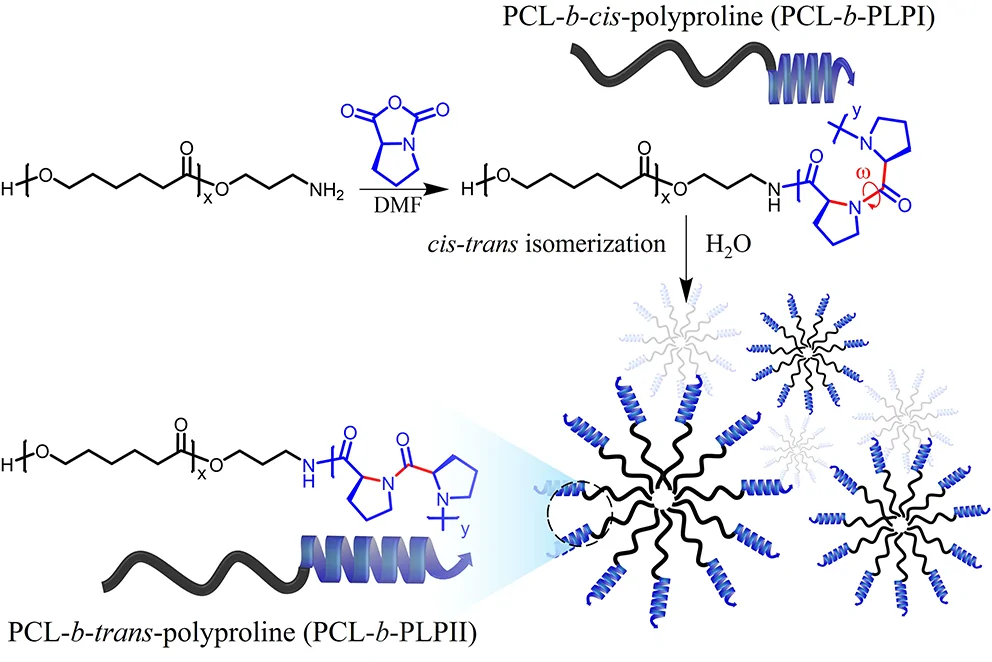
Synthetic Pathway for the Preparation of Poly­(ester–peptide) Copolymers and a Proposed Self-Assembly Mechanism to Yield Micelles Triggered by the *Cis–Trans* Polyproline Interconversion in Water

Hybrid copolymers are prepared by two-step process where a synthetic macroinitiator is first prepared with either one or two chain-ends functionalized with primary amino groups.^34^ The macroinitiator is then used to synthesize the second polypeptide domain using conventional methods of NCAs polymerization. Our strategy consisted in using PCL amino-ended (PCL-NH_2_) for triggering the Pro-NCA polymerization to produce poly­(ester-peptide) copolymers. We prepared PCL-NH_2_ of *M*_n_ = 1990 g·mol^–1^ by a two-step protocol, using Boc-aminopropanol as initiator in the bulk, ROP of ε-caprolactone to yield PCL-NH*t*-Boc. Thus, the protected amino group on this polymer was readily regenerated under acidic conditions to give an aminated polyester (Scheme S1). The chemical constitution of the PCL-NH_2_ macroinitiator was assessed by SEC and ^1^H NMR analysis (Figure 1). On the other hand, highly pure l-proline NCA (Pro-NCA) was prepared by using *N*-*tert*-butyloxycarbonyl (*N*-Boc) protected l-proline (Figure S2). This method has allowed access to well-defined PLP homopolymers and copolymers.^14^ Thereof, the low molecular weight PCL-NH_2_ that is soluble in DMF was used to trigger the ROP of Pro-NCA to produce a series of poly­(caprolactone)-*b*-poly­(l-proline) (PCL_*x*_-*b*-PLP_*y*_) copolymers covering a broad range of polyproline compositions. As a control experiment, homopolyproline (*M*_n_ = 5230 g·mol^–1^) was also prepared using hexylamine as initiator (Table 1, entry 6, and Figure S3). The NCA polymerization was monitored by FTIR, and after 6 days, disappearance of NCA-carbonyl bands at 1789 and 1850 cm^–1^ indicated complete reactions. Yields between 39% and 66% were obtained for the copolymers after their recovering by precipitation in diethyl ether (Table 1, entries 2–5). The entire synthesis was also followed by SEC and ^1^H NMR. For instance, the SEC trace in CHCl_3_ of the PCL_73_-*b*-PLP_27_ copolymer displayed a clean peak shifted to a higher molecular weight region after the polypeptide copolymerization, confirming the successful synthesis (Figure 1a). Furthermore, the NMR spectra exhibited characteristic signals from both polyester and polypeptide blocks suggesting a diblock architecture (Figure 1b). Although copolymers with a PLP composition exceeding 27% were insoluble in CHCl_3_ or HFIP and could not be analyzed by SEC, their chemical structures were confirmed by NMR spectra recorded in a CHCl_3_/TFA-*d* mixture (Figure S4).

**Figure 1:**
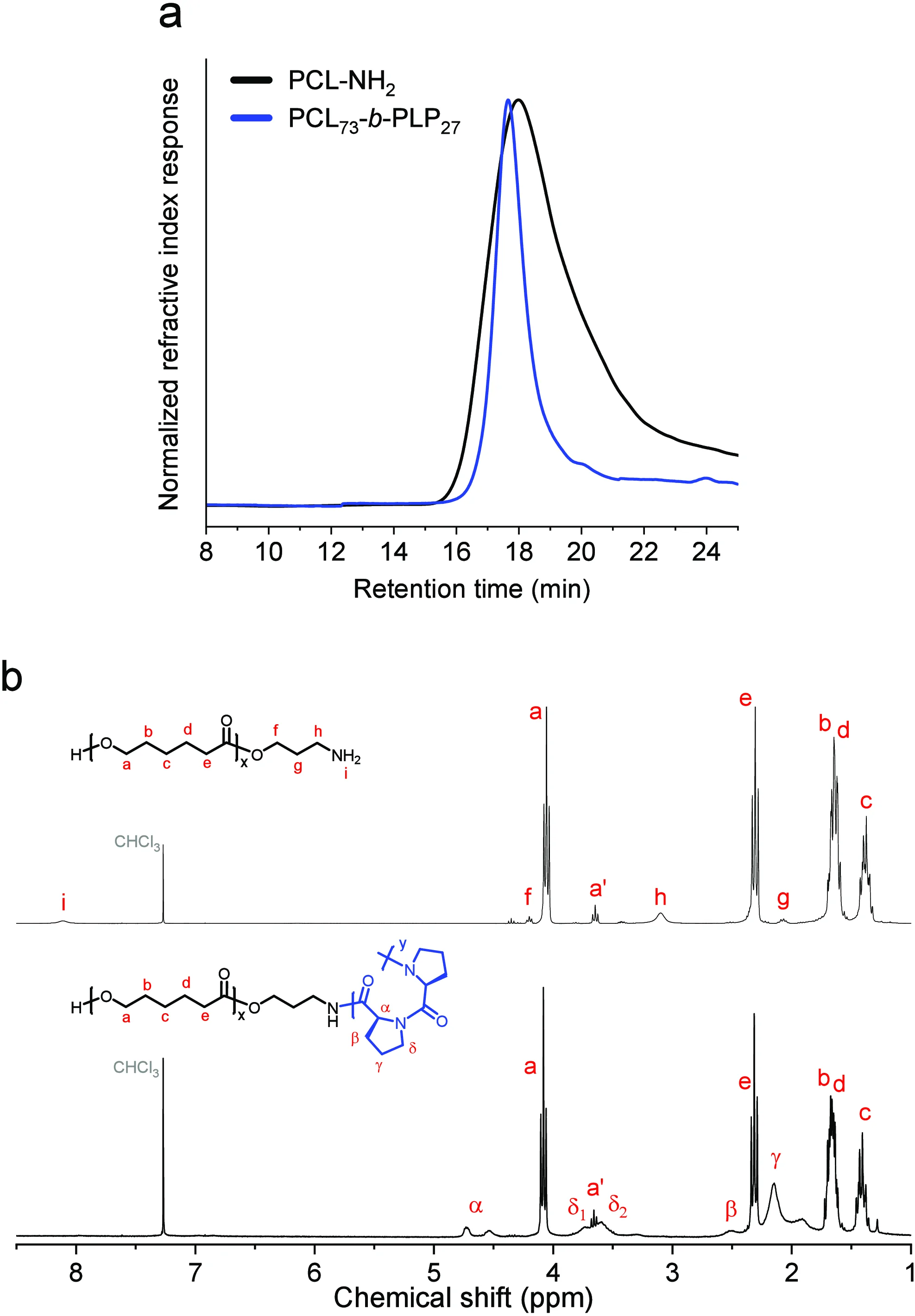
(a) SEC traces and (b) ^1^H NMR (in CDCl_3_) spectra of PCL-NH_2_ and the PCL_73_-*b*-PLP_27_ copolymer.

**Table 1:** Characterization of the Poly­(ε-caprolactone)_*x*_-*b*-poly­(l-proline)_*y*_ (PCL_*x*_-*b*-PLP_*y*_) Copolymers

entry	polymer^a^	yield^b^ (%)	molar composition^c^ [PCL]/[PLP]	*M*_n_ ^c^ (g·mol^–1^)	*M*_n_ ^d^ ^,^ [Table-fn t1fn5] (g·mol^–1^)	*Đ* ^d^ ^,^ [Table-fn t1fn5]
1	PCL-NH_2_	80	100/0	1990	2640	1.41
2	PCL_73_-*b*-PLP_27_	39	73/27	2640	2990	1.23
3	PCL_33_-*b*-PLP_67_	62	33/67	5455		
4	PCL_17_-*b*-PLP_83_	66	17/83	10360		
5	PCL_5_-*b*-PLP_95_	62	5/95	31600		
6	PLP^f^	83	0/100	5230		

a Subscripts correspond to the molar compositions of each block in the copolymer.

b Isolated yield after precipitation and drying.

c Molar composition and number–average molecular weight as determined by ^1^H NMR.

d From SEC: SEC experiments were performed with chloroform.

f Polymers from entries 3–6 are neither chloroform- nor HFIP-soluble.

g As a control experiment, poly­(l-proline) was synthesized in DMF using hexylamine as the initiator at a monomer-to-initiator feed ratio of [M]/[I] = 50.

The thermal behavior of the copolymers as well as their corresponding parent polymers (PCL-NH_2_ and PLP) was explored by TGA, DSC and POM to further disclose their thermal stability, thermal transitions and crystalline morphology, respectively. Thermal transitions are collected in Table S2. The thermogravimetric traces of homopolymers and copolymers recorded under inert atmosphere in the 50–600 °C range are shown in Figure 2a and a selection of their derivatives curves in Figure S5. PCL-NH_2_ and PLP started to decompose at 303 °C and 210 °C, respectively, and showed one and two degradation steps, respectively. Interestingly, in all copolymers the decomposition progressed in three steps, where each step corresponded to its parent polymer and at a rate that is closely related on the content of prolyl units with practical no residual left at 600 °C. The DSC traces of homopolymers and copolymers recorded at heating and cooling over the 25–180 °C range are depicted in Figure 2b, and the second heating is found in Figure S6. Only PCL and copolymers with a minimum of 33% of caprolactone units exhibited thermal transitions distinctively of melting and crystallization which are attributed to the polyester block. It is noteworthy, however, that in copolymers with a minimal of 67% prolyl units a broad thermal transition with a maximum centered at 60–80 °C arouse indicative of the presence of the polypeptide block which disappeared in the second heating. Thus, such thermal event can be associated not to a melting but to an intramolecular rearrangement of prolyl residues. Since nitrogen in proline residues bears no amide hydrogen, making impossible any hydrogen bond, PLPI and PLPII are the predominantly detectable conformations.^35,36^ This thermal transition, however, is not surprising since polypeptides tend to undergo conformational, phase transition changes in secondary structures that are sensitive to temperature.^37^ Together, these thermal results anticipated the existence of a microphase separation.^38,39^ In addition, POM observations revealed that copolymers with 27% of polyproline formed smaller spherulites than the parent PCL precursor, suggesting a strong repressing effect that prolyl residues exerted on the genuinely crystallinity of PCL (Figure S7).

**Figure 2:**
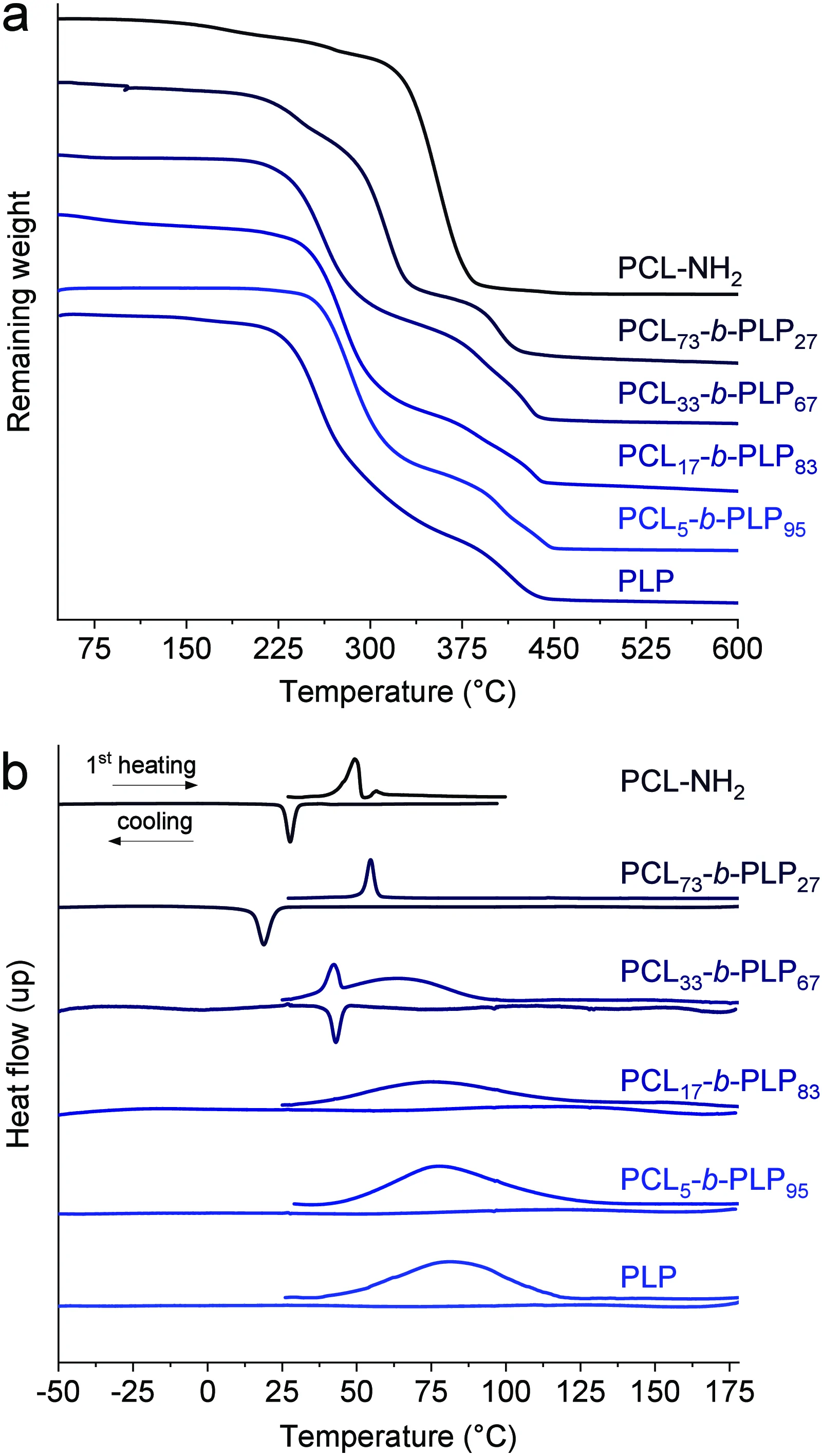
(a) TGA and (b) DSC (first heating and cooling) traces of the PCL_*x*_-*b*-PLP_*y*_ copolymers.

Then, we explored the capacity of the polyproline-based copolymers to self-assemble in water. As we used DMF as reaction medium to marry PCL to PLP, and such solvent favors the synthesis of all-*cis*, right-handed PLPI, we hypothesized that redissolving the copolymer by simple stirring in water would induce the all-*trans*, left-handed PLPII conformation and a steadily microphase separation. Thus, a spontaneous self-assembling will occur due to the *cis–trans* isomerization about the peptide bonds which provokes copolymer solubilization. We used DLS and OP measurements to firstly disclose our approach. Table 2 collects results of DLS and specific rotations of all copolymers and PLP homopolymer. We prepared copolymer dispersions in water (1.9 mg·mL^–1^) under magnetic stirring to let them progressively redissolved and yielded nanoaggregates of *z*-average diameters ranging from 162 to 103 nm and PDI between 0.29–0.35. The size of the nanoparticles followed a trend, decreasing as the polypeptide block length increase (Figure S8). Positive zeta potentials were obtained but without showing a recognizable trend.

**Table 2:** Solution Properties of Polyproline-Based Copolymers: *z*-Average Diameter, Polydispersity, Zeta Potential and Specific Rotations

polymer	*z*-average diameter^a^ (nm)	PDI^a^	ζ^a^ (mV)	[α]_D_^25^ ^b^
PCL_73_-*b*-PLP_27_	162 ± 1.5	0.30	14.5	
PCL_33_-*b*-PLP_67_	142 ± 6.0	0.39	15.0	–316
PCL_17_-*b*-PLP_83_	106 ± 3.5	0.29	12.7	–371
PCL_5_-*b*-PLP_95_	103 ± 1.8	0.35	14.7	–434
PLP				–524

a Obtained from the DLS measurements.

b Specific rotation measured in water at 25 °C.

Encouraged by the DLS results, we freeze-dried all polymer solutions, recorded FTIR spectra, and compared them to pristine copolymer samples to shed light on the conformation adopted by prolyl residues (Figure S9). Infrared bands at 1,355 and 960 cm^–1^ are typical of PLPI,^40^ after self-assembling, such bands in copolymers exceeding 27% of prolyl residues were absent and their infrared spectra were identical to the PLPII spectrum. These spectroscopic results were a first hint that polyproline mutarotated into polyproline II. To better comprehend the mutarotation phenomenon, we collected specific rotations by OP measurements using identical copolymer concentrations at designated time intervals (Table 2 and Figure 3a).

**Figure 3:**
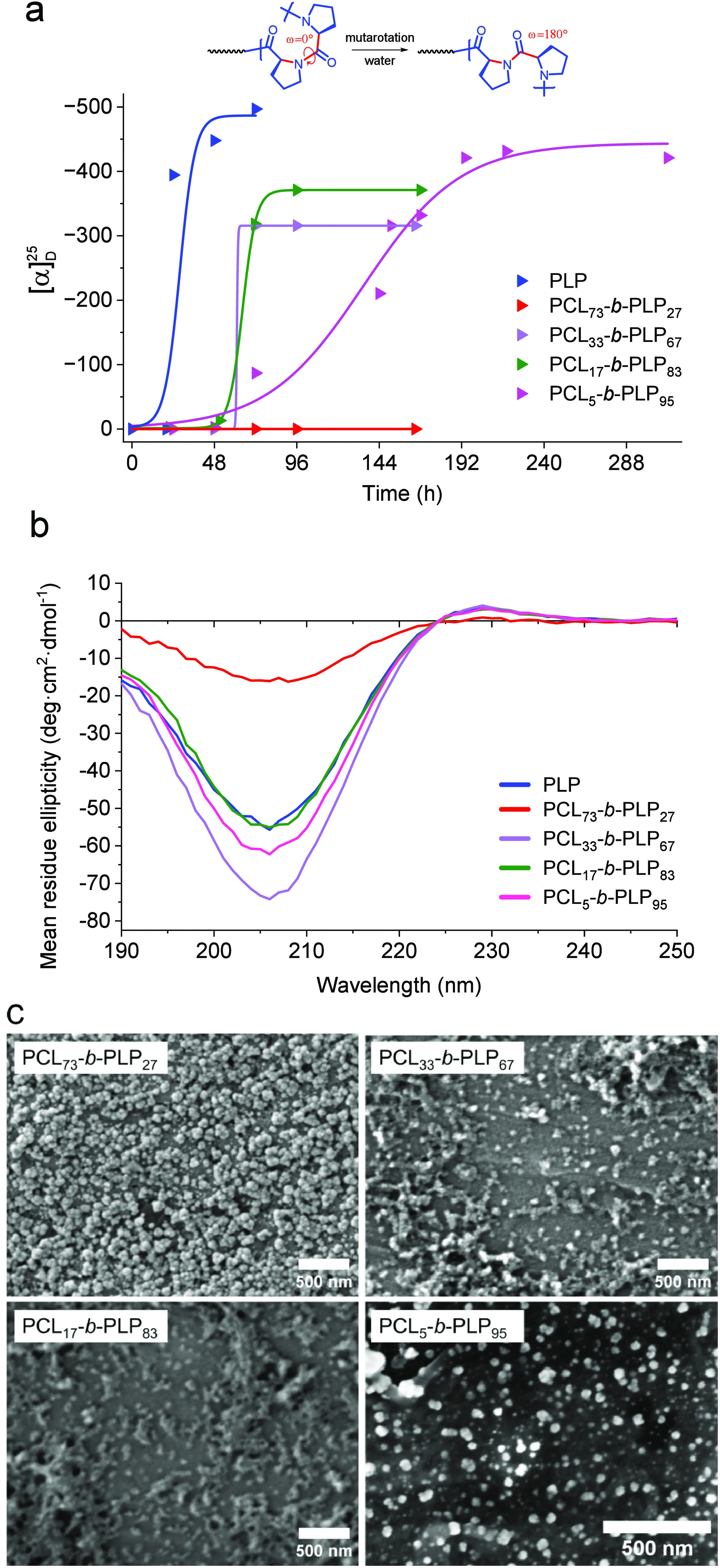
(a) Specific rotations, (b) CD spectra and (c) SEM images (scale bar = 500 nm) of the polyproline-based copolymers.

As control experiment, we first monitored the mutarotation of PLP homopolymer, and in 3 days, it became soluble showing a specific rotation of [α]_D_^25^ = −524°.^41^ Then, copolymers with a polyproline composition between 95% and 67% exhibited steady specific rotations between [α]_D_^25^ = −434° and [α]_D_^25^ = −316° in 9 days, obtaining transparent copolymer solutions (Figure S10). The presence of the polyester, nonetheless, seems to repress the free prolyl mutarotation, and the copolymers required several hours to reach invariable specific rotations. Although specific rotation decreases alongside polyproline content, no specific rotation was detected for the copolymer containing only 27% polypeptide. This is not surprising since this copolymer had low PLP content and was not fully soluble.

Next, we inspected the preferential secondary structure of PLP and PLP-based copolymers by CD spectroscopy on the same solutions (Figure 3b). As reference, we first measured the PLP homopolymer and observed a strong minimum at 206 nm and a weak maximum at 225 nm which are genuine signals related to the left-handed 3-fold helix.^42,43^ As expected, copolymers with at least 67% of polyproline replicated such bands confirming the preferential all-*trans*, left-handed helices in the copolymer that sustain polyester chains in solution.

The copolymer with 27% of prolyl residues, exceptionally, displayed a weak ellipticity and could be consequence of the short polypeptide chain that restricted complete solubility. Thereof, and having confirmed that the *cis–trans* isomerization of polyproline triggered the copolymer solubilization and self-assembly, we explored the morphology of the nanoaggregates by SEM (Figure 3c) and TEM over a representative copolymer (Figure S11). A spherical morphology across all colloidal solutions was observed, with diameters that were in good agreement with the DLS values. Based on the AB diblock architecture, we propose that several unimers hierarchically associate in water to form core-shell micelles, in where the corona is made of all-*trans*, left-handed PLP helices and polycaprolactone chains constitute the core. To strengthen this hypothesis, the micellization process was examined, and the critical micelle concentration (CMC) was determined by measuring the variation in light scattering intensity with polymer concentration in water (Figure S12). As expected, plots indicated a sharp change in slope of the straight line as the concentration increased, thus, several unimers had associated to form nanoaggregates. In addition, we collected ^1^H NMR spectra in deuterated water to steadily track the solubilization of the PCL_5_-*b*-PLP_95_ copolymer (Figure S13). After 4 days a transparent solution was obtained and only prolyl signals were detected. Remarkably, PCL signals were absent, suggesting that polyester chains were shielded within the core behaving like a solid and then not appearing in the spectra.

Finally, as a proof of concept, doxorubicin hydrochloride, an antineoplastic drug widely used to treat diverse types of cancer, was encapsulated. We mixed the copolymer with 83% of PLP and the drug by stirring in water for 4 days and then dialyzed to get rid of free drug. After encapsulation, an increase in hydrodynamic diameter was observed (Figure S14). To ascertain the amount of drug, loaded micelles were dissolved in TFE to avoid any self-assembly event and analyzed by UV-vis, achieving a DLE of 31%.

In summary, we prepared a series of poly­(ester-peptide) copolymers via l-proline NCA polymerization initiated by amino-ended PCL in DMF, whose chemical constitution and thermal behavior was assessed by spectroscopic and thermal analyses, respectively. These copolymers were used to prepare a series of nanoparticles. Thereof, we reported an unprecedented method of spontaneous copolymer self-assembly that is triggered by the *cis–trans* isomerization of polyproline, promoting its solubilization to produce hybrid micelle nanoparticles. The approach reported in this Letter is conceptually different from others such as the solvent-exchange method that requires the use of toxic solvents or surfactants to prepare nanoparticles.^44,45^

The utilization of polyproline type II covalently bonded with PCL allowed us to design amphiphiles that can be promising precursors as drug nanocarriers. In fact, these nanodevices were capable of encapsulating an amphiphilic drug, doxorubicin hydrochloride, highlighting their potential as drug delivery systems. Further efforts would be oriented to optimize the encapsulation process to achieve higher encapsulation efficiencies and to explore the in vitro drug release kinetics, for instance, by changes in temperature as an external stimulus.^20^

Thus, we believe that using the genuinely characteristic of polyproline as a molecular switch along with other polymers can pave the way to extend its use in the designing of innovative peptidomimetics with promise as drug delivery systems or in other nanotechnology fields.

## Supplementary Material



## Notes

Material and methods; SEC and ^1^H NMR spectra of PCL-NH*t*-Boc, Pro-NCA; ^1^H NMR proline-based copolymers; FTIR spectra of Pro-NCA and polyproline-based copolymers; Thermal properties and TGA derivative curves and DSC traces of the second heating in copolymers; POM images of PCL-NH_2_ and copolymers; Still photos of the mutarotation of copolymer in water; TEM images; DLS profiles and correlation functions of the polyproline-based nanoparticles; Critical micelle concentration plots of copolymers; UV-vis spectra and DLS profiles of drug encapsulation; Additional references (PDF)

## Abbreviations

SEC: Size exclusion chromatography
NMR: Nuclear magnetic resonance
TGA: Thermogravimetric analysis
DSC: Differential scanning calorimetry
DLS: Dynamic light scattering
POM: Polarized optical microscopy
OP: Optical polarimetry
CD: Circular dichroism spectroscopy
SEM: Scanning electron microscopy
TEM: Transmission electron microscopy
UV-vis: Ultraviolet-visible spectroscopy
DMF: *N*,*N*-dimethylformamide
HFIP: 1,1,1,3,3,3-hexafluoro-2-propanol
TFE: 2,2,2-trifluoroethanol
DLE: Drug loading efficiency
