# Supplementary material for: Copolymer Self-Assembly Triggered by Cis–Trans Polyproline Isomerization
Source: ACS Macro Lett. 2026 Sep 3;15(9):1305–10. doi: 10.1021/acsmacrolett.6c00340 (PMC13585134; doi:10.1021/acsmacrolett.6c00340)
Supplement: Supplementary file 1 [file mz6c00340_si_001.pdf]

Copolymer Self-Assembly Triggered by *Cis-Trans* Polyproline Isomerization

Ernesto Tinajero-Díaz* and Antxon Martínez de Ilarduya

Departament d´Enginyeria Química, Universitat Politècnica de Catalunya,

ETSEIB, Diagonal 647, 08028, Barcelona, Spain.

E-mail: ernesto.tinajero@upc.edu

Supporting Information

Contents

1. Materials......2

2. General methods......2

2.1 Synthesis of amino-ended poly(ε-caprolactone) ($PCL-NH_2$)......4

2.2 Synthesis of L-proline *N*-carboxyanhydride (Pro-NCA)......5

2.3 Synthesis of poly(ε-caprolactone)$_x$-*block*-poly(L-proline)$_y$ (PCL_x-*b*-PLP_y) copolymers......6

2.4 Loading of doxorubicin hydrochloride (DOX·HCl) in the PCL_x-*b*-PLP_y copolymers......6

3. Figures, schemes and tables......6

Scheme S1. Synthesis of amino-terminated poly(ε-caprolactone) ($PCL-NH_2$).[1]......6

Table S1. Characterization of the boc-amino protected poly(ε–caprolactone) (PCL-NH*t*-Boc) and amino-terminated poly(caprolactone) ($PCL-NH_2$)......7

Figure S1. (a) SEC trace and (b) ^{1}H NMR ($CDCl_3$, 300 MHz) spectra of the Boc-amino protected PCL (PCL-NH*t*-Boc) and amino-ended PCL ($PCL-NH_2$)......7

Figure S2. (a) ^{1}H, (b) ^{13}C NMR spectra ($CDCl_3$, 300 MHz) and (c) ATR-FTIR spectrum of L-proline NCA......8

Figure S3. ^{1}H NMR ($CDCl_3$/TFA-d (85:15 v/v, 300 MHz) spectrum of homopolyproline. * = Traces of solvent, i.e. DMF......8

Figure S4. ^{1}H NMR ($CDCl_3$/TFA-d (85:15 v/v, 300 MHz) spectra of the PCL_x-*b*-PLP_y copolymers...9

Table S2. Thermal properties of the PCL_x-*b*-PLP_y copolymers......9

Figure S5. A selection of TGA derivative curves of the PCL_x-*b*-PLP_y copolymers......10

Figure S6. DSC traces of the second heating for polymer parent and all PCL_x-*b*-PLP_y copolymers...10

Figure S7. POM micrographs of $PCL-NH_2$ and PCL_{73}-*b*-PLP_{27} showing the spherulites obtained after isothermal crystallization cooling from the melt at 35 ºC......11

Figure S8. DLS profiles and correlation functions of the PCL_x-*b*-PLP_y copolymers......11

Figure S9. FTIR spectra of: (a) PCL_x-*b*-*cis*-PLP_y and (b) PCL_x-*b*-*trans*-PLP_y copolymers......12

Figure S10. Still photos displaying the effect of the polyproline block mutarotation on the turbidity of the PCL_{33}-*b*-PLP_{67} solution in water......12

Figure S11. A selection of TEM images of nanoparticles from the PCL_{17}-*b*-PLP_{83} copolymer. Scale bar = 100 nm......13

Figure S12. Critical micelle concentration of selected polyproline-based copolymers......13

Figure S13. (a) ^{1}H NMR spectra (D_2O, 300 MHz) of the PCL_5-*b*-PLP_{95} diblock copolymer recorded at different time points. (b) Still photo of the NMR tube exhibiting the fully soluble copolymer after 96 h. 14

Figure S14. Drug encapsulation in polyproline–based nanoparticles. (a, b) UV-vis spectra of DOX·HCl standards in TFE and the corresponding calibration curve. (c, d) DLS profiles (inset: correlation coefficient) and UV-vis spectra of blank and loaded nanoparticles prepared from the PCL_{17}-*b*-PLP_{83} copolymer. 14

4. References 15

1. Materials

3-(Boc-amino)-1-propanol (95%), Boc-L-proline (97%), epichlorohydrin (ECH) (>99.0%) and triphosgene (99%) were acquired from Fluorochem Ltd (U.K.). ε-Caprolactone (97%), tin(II) 2-ethylhexanoate ($Sn(Oct)_2$), doxorubicin hydrochloride (DOX·HCl) (98.0-102.0%), *N,N*-dimetilformamida (≥99.8%, anhydrous), acetonitrile (ACN) (>99.9%), tetrahydrofuran (THF) (>99.9), ethyl acetate (>99.5%), trifluoroacetic acid (TFA) (>99.0%) and 2,2,2-trifluoroethanol (TFE) (≥99%) were acquired from Sigma Aldrich. Chloroform-*d* ($CDCl_3$) and deuterium oxide (D_2O) were purchased from Euroisotop. Unless otherwise noted, all reagents and chemicals were used as received without further purification.

2. General methods

^{1}H and ^{13}C NMR spectra were recorded on a Bruker AMX-300 spectrometer at 25 ºC, operating at 300.1 and 75.5 MHz, respectively. Compounds were dissolved in deuterated chloroform or a deuterated chloroform/trifluoroacetic acid mixture (85:15) ($CDCl_3$/TFA-d) or deuterated water (D_2O). All spectra were internally referenced to tetramethylsilane (TMS) or the residual signal of the solvent. About 10 and 50 mg of sample in 1 mL of solvent were used for ^{1}H and ^{13}C NMR, respectively. Sixty-four scans were recorded for ^{1}H, and between 5,000 and 20,000 scans for ^{13}C NMR.

Size exclusion chromatography (SEC) for molecular weights were determined using chloroform within a Waters equipment (Foster City, CA, USA), provided with RI and UV detectors and HR5E and HR2 Waters linear Styragel columns (7.8 mm × 300 mm). Of the sample solution, 0.1 mL (0.1% w/v) was injected and chromatographed with a flow rate of 0.5 mL·min^{-1}. The molar mass averages and distributions were calibrated against monodispersed polystyrene standards.

Fourier transform infrared spectra (FT-IR) were acquired using a Perkin-Elmer Frontier FT-IR spectrometer (Waltham, MA, USA), provided with a universal-attenuated total reflectance ATR accessory. Infrared spectra were recorded in the 4,000–600 cm^{-1} range at a resolution of 4 cm^{-1} and 16 scans were collected.

The thermal behavior of polymers was examined by differential scanning calorimetry (DSC) using a Perkin-Elmer Pyris apparatus. The thermograms were obtained from 4–6 mg samples at heating and cooling rates of 10 ºC·min^{-1} under a nitrogen flow of 20 mL·min^{-1}. Indium and zinc were used as standards for temperature and enthalpy calibration. The melting temperature was taken as the maximum of the endothermic peak arising on the heating traces recorded from samples crystallized from the melt.

Thermogravimetric analysis (TGA) was performed on a Mettler-Toledo TGA/DSC 1 Star System under a nitrogen flow of 20 mL·min^{-1} at a heating rate of 10 ºC·min^{-1} and within a temperature range of 30ꟷ600 ºC.

Dynamic light scattering (DLS) measurements used for particle hydrodynamic size and ζ-potential determinations were performed with the Zetasizer NanoSeries Malvern instrument equipped with a 4 mW He-Ne laser operated at 632.8 nm. Particles were suspended in deionized water at 25 ºC and placed in glass cuvettes. The non-invasive back-scatter optic arrangement was used in order to collect the light scattered by particles at an angle of 173º.

Scanning electron microscopy (SEM) images were taken with a field-emission JEOL JSM-7001F (JEOL, Tokyo, Japan) from platinum/palladium coated samples. Different dilutions were essayed to observe free individual nanoparticles. Images were edited using the ImageJ software.

Transmission Electron Microscopy (TEM) images were observed in the Cryoelectron Microscopy Lab of the Electron Microscopy and Related Techniques Unit from the Scientific and Technological Centers of the Universitat de Barcelona. Samples were adsorbed onto glow-discharged carbon-coated copper grids for 1 min and, then, washed with milliQ water for 1 min. They were negatively stained by floating the grid on a drop of 2% uranyl acetate for 1 min. The excess of liquid was blotted manually from the edge of the grids. The sample was observed in a JEOL-1010 electron microscope (Jeol, Japan) equipped

with a tungsten cathode. Images were acquired at 80 kV with a 1kx1k with a CCD camera SIS Megaview III.

Optical rotation measurements were performed using an electronic polarimeter (P 3001, A. Krüss Optronic, Germany) equipped with a sodium vapor lamp operating at 589 nm. Samples were prepared in water at a concentration of 1.9 mg·mL^{-1} and measured at room temperature using a 100 mm path-length polarimeter cell. Measurements were carried out in automatic mode, and the reported values represent the average of repeated readings. Specific rotation was calculated using the equation:

$$[\alpha]_D^{25} = \frac{\alpha}{l \text{ x } c}$$

where [α] is the specific rotation, l is the length of the sample tube in decimeters, and c is the concentration of the sample in grams per milliliter of solution.

Circular dichroism measurements were carried out using a Jasco spectropolarimeter J-815 (Centres Científics i Tecnològics, Universitat de Barcelona). Briefly, solutions of PCL_x-*b*-PLP_y copolymers at concentration of 10 µg·mL^{-1} in deionized water were prepared. Spectra were recorded in the 190–250 nm range using a 10 mm quartz cell at a scanning speed of 10 nm·s^{-1} at 25 °C.

Ultraviolet–visible (UV-Vis) spectroscopy. Quantification of entrapped DOX·HCl was performed on a SpectroStar Nano UV-Vis Spectrophotometer (BMG LABTECH) at a wavelength λ=486 nm, and using standards of drug in TFE.

2.1 Synthesis of amino-ended poly(ε-caprolactone) (PCL-NH_2)

The amino-ended polycaprolactone (PCL-NH_2) was synthesized via a two-step procedure (Scheme S1): (a) ROP of ε-caprolactone: in a 25 mL three-necked, round-bottom flask equipped with a mechanical stirrer, a nitrogen inlet and a vacuum distillation outlet, ε-caprolactone (5.0 g, 43.80 mmol) and 3-(Boc-amino)-1-propanol (0.45 g, 2.57 mmol) were added. The flask was then immersed in an oil bath at 100 °C under a N_2 atmosphere for 5 min for purging the reaction system. Thereof, the flask was heated up 110 °C and polymerization began after the addition of $Sn(Oct)_2$ (17.7 mg, 0.0438 mmol, 0.04% by weight relative to the lactone) from a stock solution in chloroform. The reaction flask was left under

stirring for 6 h until ε-caprolactone was fully consumed, as monitored by ^{1}H NMR. The mixture was then dissolved in $CHCl_3$ and precipitated into an excess of hexane. The copolymer was recovered as a white, fluffy powder. Prior to characterization, the polymer was dried at 50 ºC under vacuum. (b) Removal of the Boc group: PCL-NH*t*-Boc (0.50 g) was placed in a round-bottom flask and dissolved in dichloromethane (5 mL). Trifluoroacetic acid (3 mL) was added dropwise and the reaction was allowed to proceed at room temperature for 5 min. The resulting solution was then precipitated into cold diethyl ether and the polymer isolated by centrifugation. The crude product was dissolved in chloroform and successively washed with an aqueous 5% $NaHCO_3$. The organic phase was dried under reduced pressure to afford the aminated polymer.

PCL-NH*t*-Boc: ^{1}H NMR ($CDCl_3$, 300 MHz): δ 4.14 (t, 2H), 4.06 (t, 2H), 3.65 (t, 2H), 3.20 (q, 2H). 2.31 (t, 2H), 1.81 (quint, 2H), 1.65 (m, 4H), 1.44 (s, 9H), 1.39 (m, 2H).

PCL-NH$_2$: ^{1}H NMR ($CDCl_3$, 300 MHz): δ 8.09 (b, 2H) 4.22 (t, 2H), 4.06 (t, 2H), 3.65 (t, 2H), 3.11 (b, 2H). 2.31 (t, 2H), 2.08 (quint, 2H), 1.65 (m, 4H), 1.39 (m, 2H).

2.2 Synthesis of L-proline *N*-carboxyanhydride (Pro-NCA).

To a round-bottom flask, *N*-Boc-L proline (5 g, 23.2 mmol, 1.0 eq), acetonitrile (75 mL), epichlorohydrin (8.6 g, 92.9 mmol, 4 eq.) were added under magnetic stirring. Afterwards, the flask was immersed in an ice bath and triphosgene (3.21 g, 11.6 mmol, 0.5 eq) was added in one portion. The reaction mixture was let to react 3 h before the filtration into a two-neck bottom flask. Then, ¾ of the ACN was removed under reduced pressure. Afterwards, and with a syringe, the mixture was precipitated in 100 mL of cold hexane under strong stirring and kept overnight in freezer. Hexane was removed under reduced pressure and 15 mL of ethyl acetate was added to the remaining solution. Thereafter, the solution was precipitated into cold hexane (4-fold excess) by using a syringe and let it to precipitate overnight in freezer. Subsequently, hexane was removed by using a syringe and Pro-NCA was redissolved in 15 mL of ethyl acetate, and it was precipitated in 100 mL of cold hexane. This process was repeated thrice. Finally, the purified Pro-NCA was dried overnight in a desiccator and kept sealed at -20 °C. Yield: 65%.

Pro-NCA: ^{1}H NMR ($CDCl_3$, 300 MHz): δ 4.34 (dd, 1H), 3.77 (dt, 1H), 3.32 (ddd, 1H), 2.28 (m, 1H), 2.27 – 2.06 (m, 2H), 1.95 (m, 1H). ^{13}C-NMR ($CDCl_3$,75.5 MHz,): δ 168.7, 154.8, 63.0, 46.4, 27.5, 26.8.

2.3 Synthesis of poly(ε-caprolactone)$_x$-*block*-poly(L-proline)$_y$ (PCL$_x$-*b*-PLP$_y$) copolymers

For the synthesis of PCL$_{73}$-*b*-PLP$_{27}$ (targeted PCL:PLP molar ratio = 2:1), Pro-NCA (52 mg, 0.37 mmol) was dissolved in anhydrous DMF (5 mL) in a Schlenk tube under an inert atmosphere. Then a solution of PCL-NH_2 (87 mg, 0.043 mmol) in dried DMF was injected into the Schlenk tube through a septum with a syringe under nitrogen atmosphere. The reaction was left under stirring until the Pro-NCA was completely consumed as monitored by FTIR spectroscopy. Then the copolymer was precipitated into an excess of diethyl ether, recovered by centrifugation and dried under vacuum. Yield: 39%.

2.4 Loading of doxorubicin hydrochloride (DOX·HCl) in the PCL$_x$-*b*-PLP$_y$ copolymers.

For loading of DOX·HCl within the core-shell micelles, 80 µg of drug and 6.2 mg of PCL$_{17}$-*b*-PLP$_{83}$ copolymer were mixed in 3.5 mL of ultrapure water under magnetic stirring in darkness for four days. Afterwards, the mixture was dialyzed (MWCO=6.0 kDa) 24 h against deionized water to remove unloaded drug. The loaded micelles were characterized by DLS and thereof, the sample was freeze-dried. Then, the loaded micelles were dissolved in TFE to avoid any self-assembly event and the amount of entrapped DOX·HCl was determined by measuring the absorbance at 486 nm. The drug-loading efficiency (DLE) was estimated using the following equation:

$$\mathrm{DLE\%} = \frac{\text{Mass of the drug in nanoparticles}}{\text{Mass of the drug in feed}} 100$$

3. Figures, schemes and tables

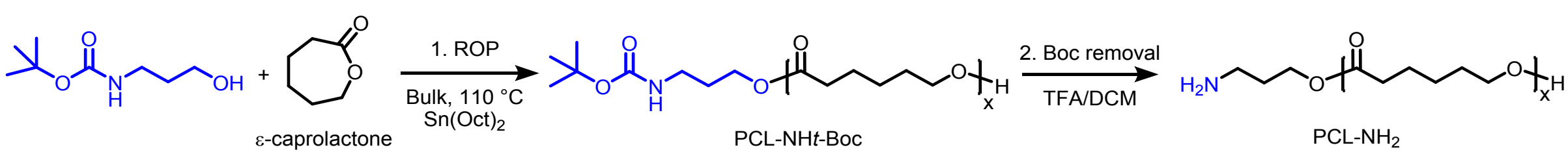

Scheme S1. Synthesis of amino-terminated poly(ε-caprolactone) (PCL-NH_2).[1]

Table S1. Characterization of the boc-amino protected poly(ε–caprolactone) (PCL-NH*t*-Boc) and amino-terminated poly(caprolactone) (PCL-NH_2).

**Polymer**	**Yield** [a] **(%)**	M_n [b] **(g·mol^{-1})**	M_n [c] **(g·mol^{-1})**	*Đ* [c]
PCL-NH*t*-Boc	96	1,870	2,780	1.31
PCL-NH_2	80	1,990	2,640	1.41

[a] Isolated yield, after precipitation and drying. [b] Number average molecular weight as determined by ^{1}H NMR ($CDCl_3$). [c] From SEC. SEC experiments were performed in chloroform.

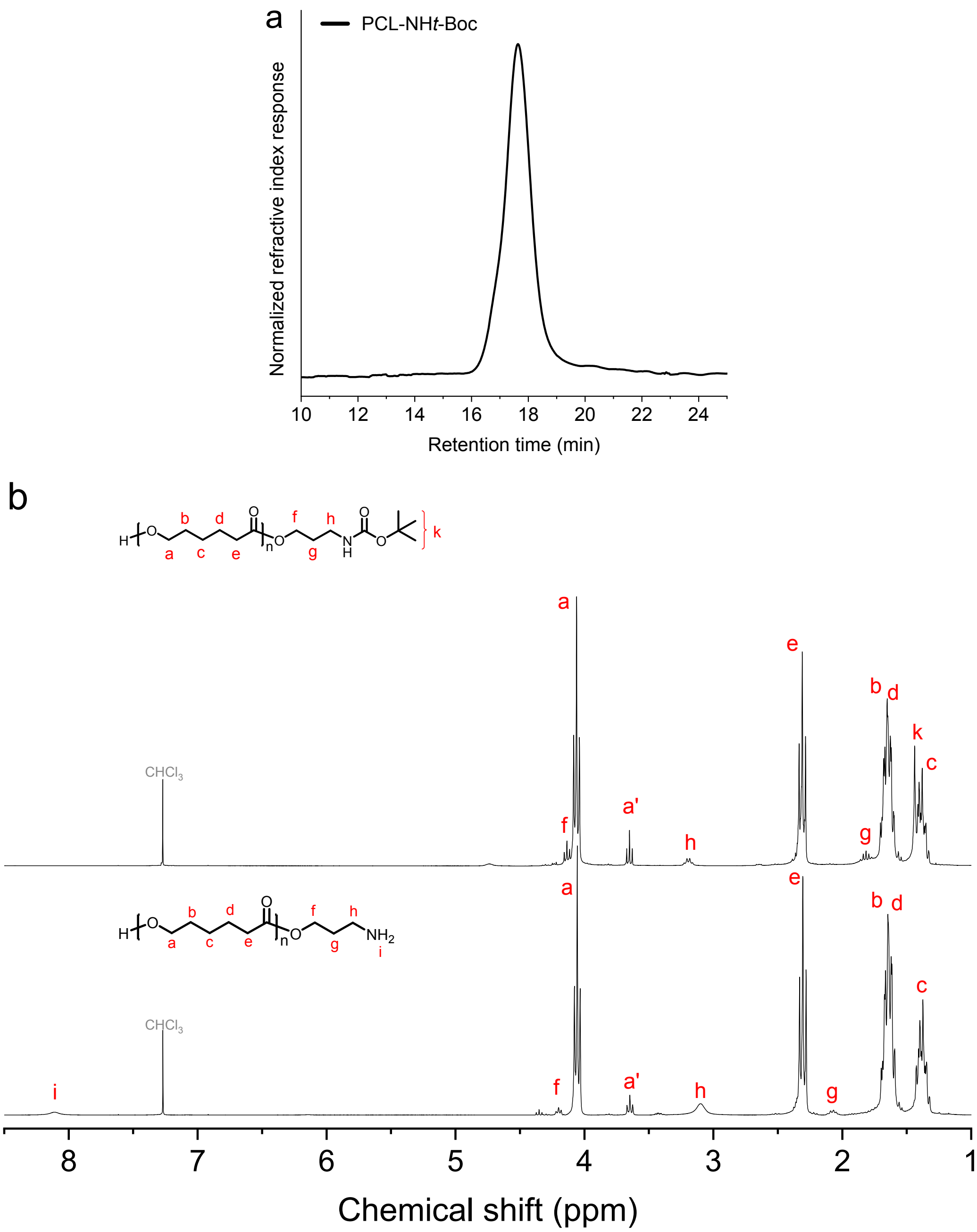

Figure S1. (a) SEC trace and (b) ^{1}H NMR ($CDCl_3$, 300 MHz) spectra of the Boc-amino protected PCL (PCL-NH*t*-Boc) and amino-ended PCL (PCL-NH_2).

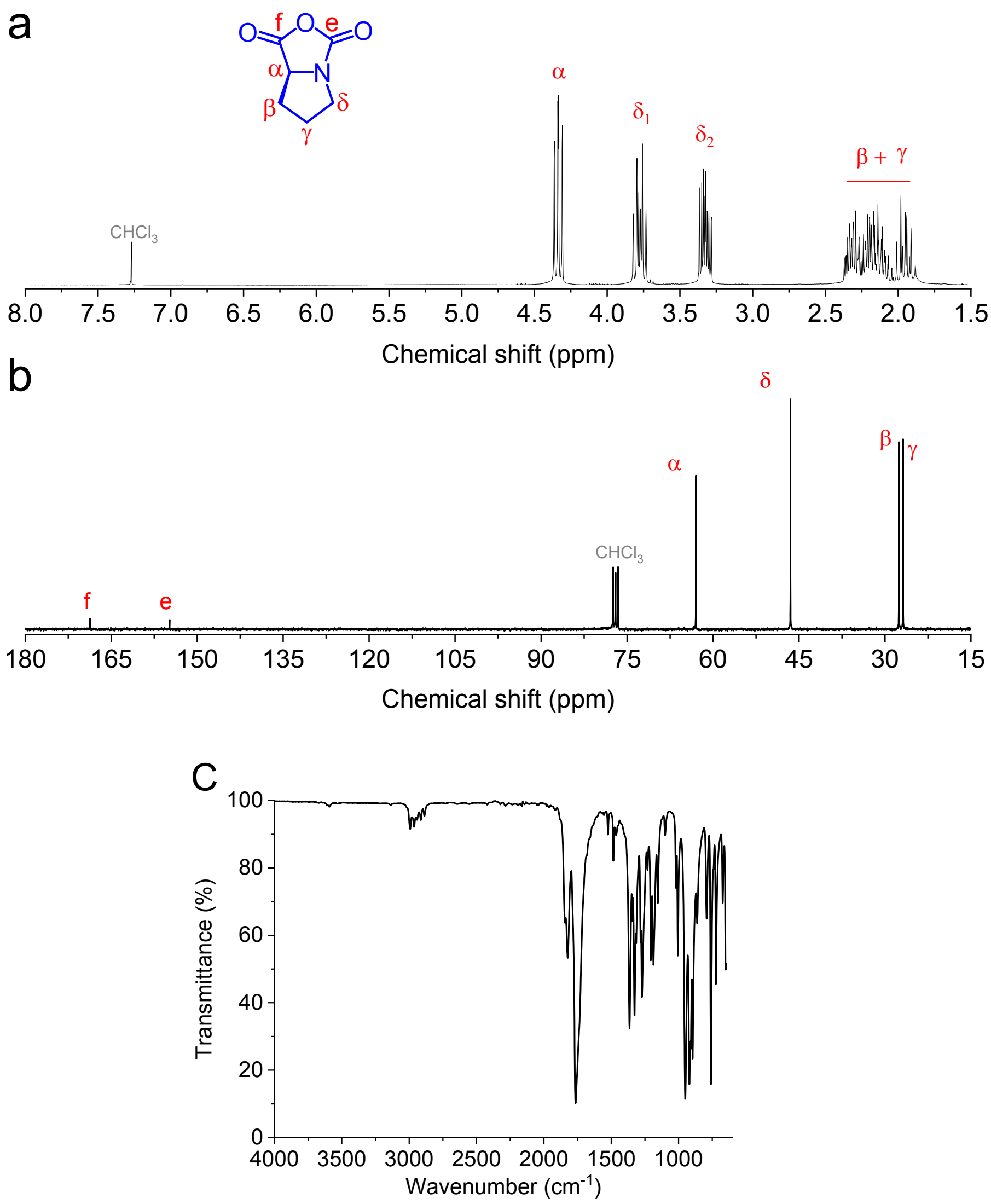

Figure S2. (a) ^{1}H, (b) ^{13}C NMR spectra ($CDCl_3$, 300 MHz) and (c) ATR-FTIR spectrum of L-proline NCA.

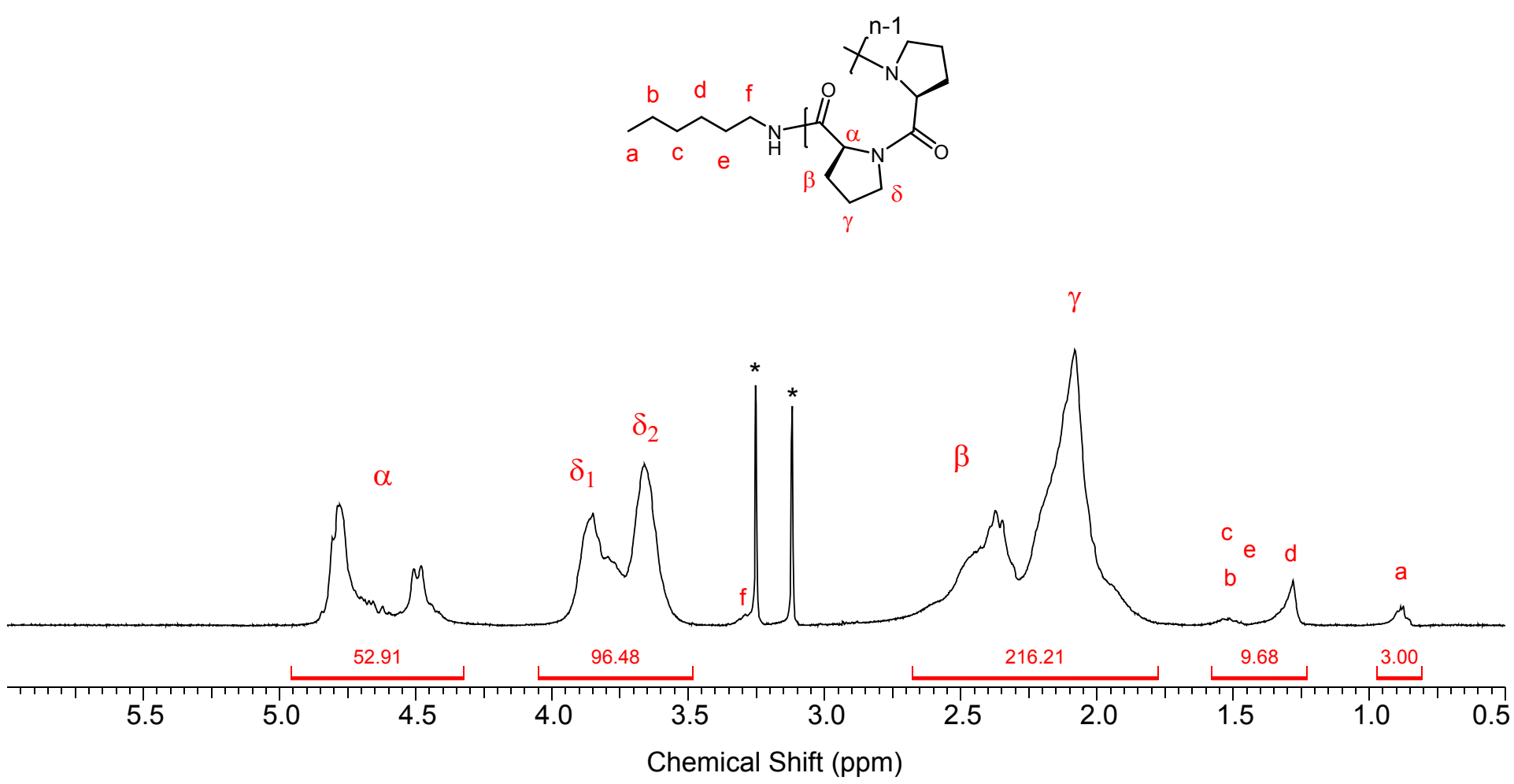

Figure S3. ^{1}H NMR ($CDCl_3$/TFA-d (85:15 v/v, 300 MHz) spectrum of homopolyproline. * = Traces of solvent, i.e. DMF.

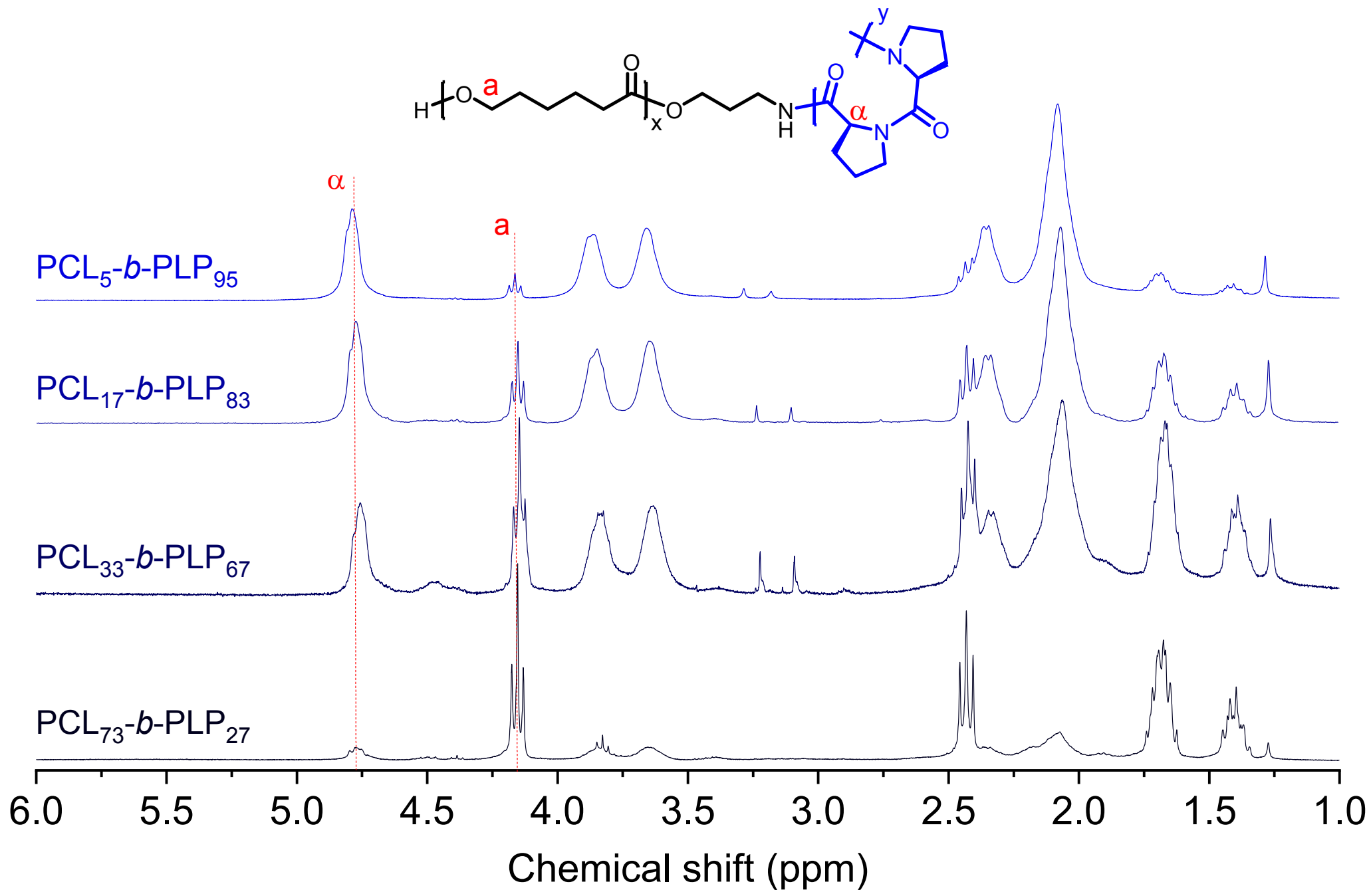

Figure S4. ^{1}H NMR ($CDCl_3$/TFA-d (85:15 v/v, 300 MHz) spectra of the PCL_x-*b*-PLP_y copolymers.

Table S2. Thermal properties of the PCL_x-*b*-PLP_y copolymers.

Polymer	TGA			DSC					
				1st heating		Cooling		2nd heating	
	$^{o}T_d$ °C	$^{max}T_d$ °C	R_w %	T_m °C	ΔH_m J·g^{-1}	T_c °C	ΔH_c J·g^{-1}	T_m °C	ΔH_m J·g^{-1}
PCL-NH_2	265	354	0.5	49	81.1	27	-71.8	43	73.8
PCL_{73}-*b*-PLP_{27}	236	313	0.0	54	60.5	28	-71.4	45	29.4
PCL_{33}-*b*-PLP_{67}	237	260	3.1	43/65	3.7/6.1	42	-1.2	57	0.81
PCL_{17}-*b*-PLP_{83}	241	276	0.0	75	14.8	-	-	-	-
PCL_5-*b*-PLP_{95}	140	286	0.0	78	39.7	-	-	-	-
PLP	233	256	0.0	82	42.5	-	-	-	-

[a] Onset temperature for 10% of weight loss ($^{o}T_d$), maximum rate ($^{max}T_d$) decomposition temperatures and remaining weight (R_w). Crystallization (T_c and ΔH_c) and melting (T_m and ΔH_m) temperatures and enthalpies measured by DSC.

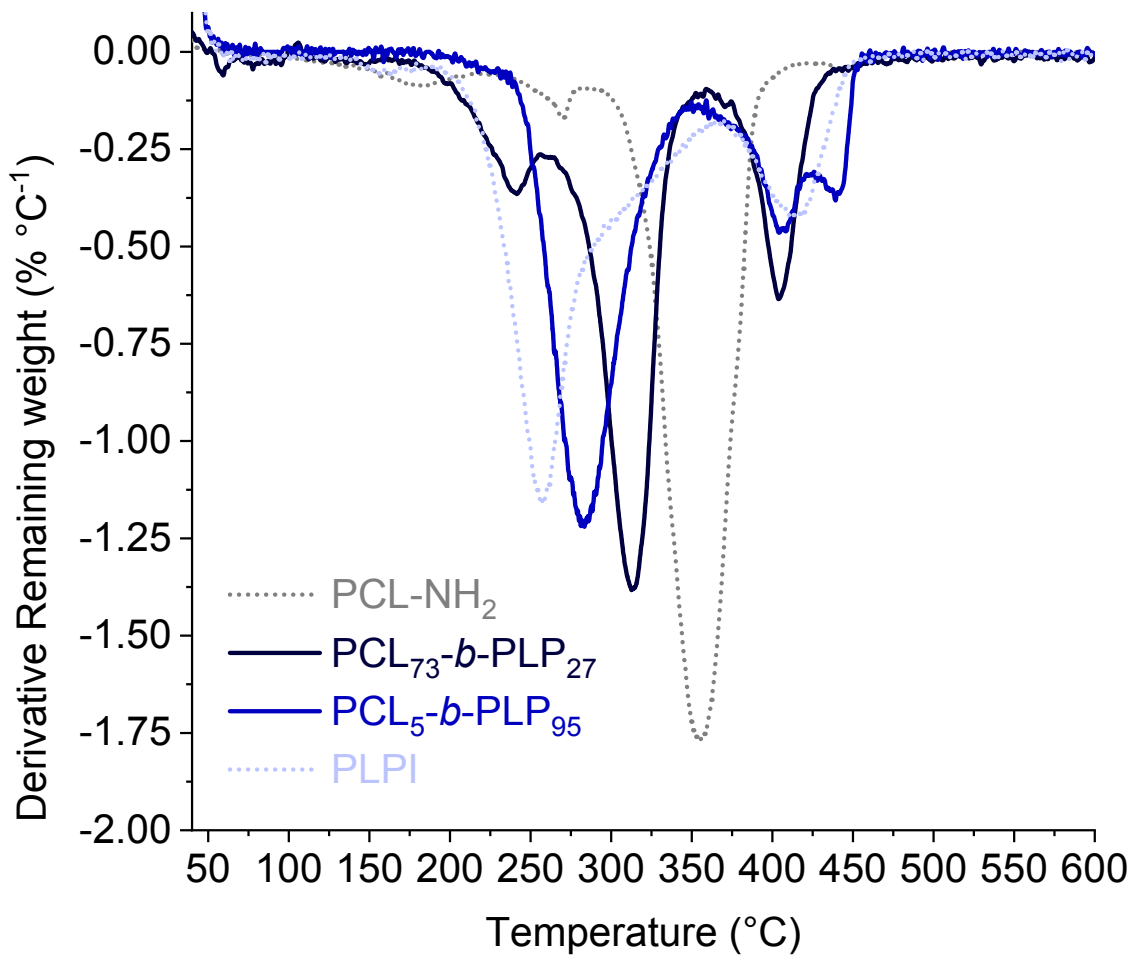

Figure S5. A selection of TGA derivative curves of the PCL_x-*b*-PLP_y copolymers.

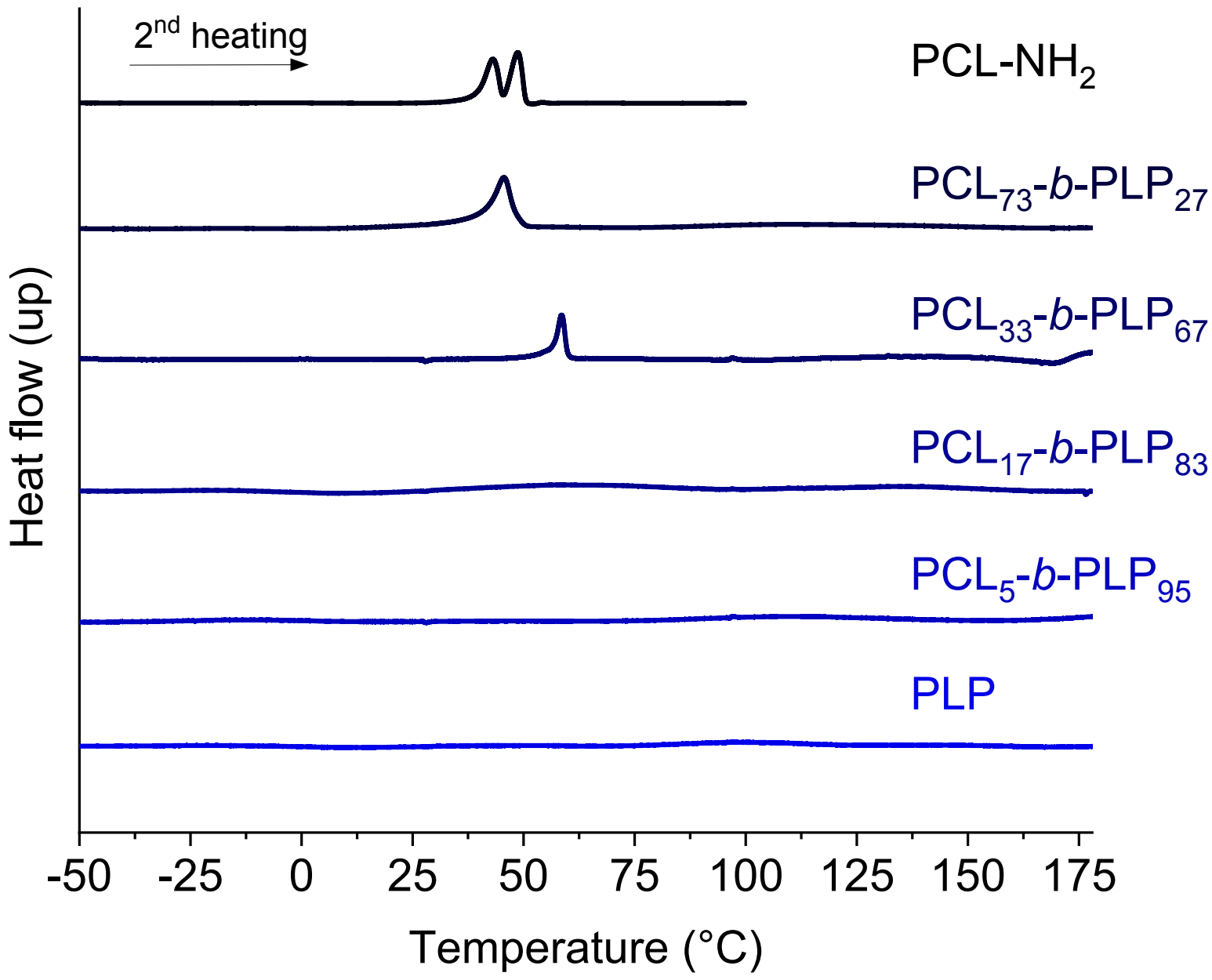

Figure S6. DSC traces of the second heating for polymer parent and all PCL_x-*b*-PLP_y copolymers.

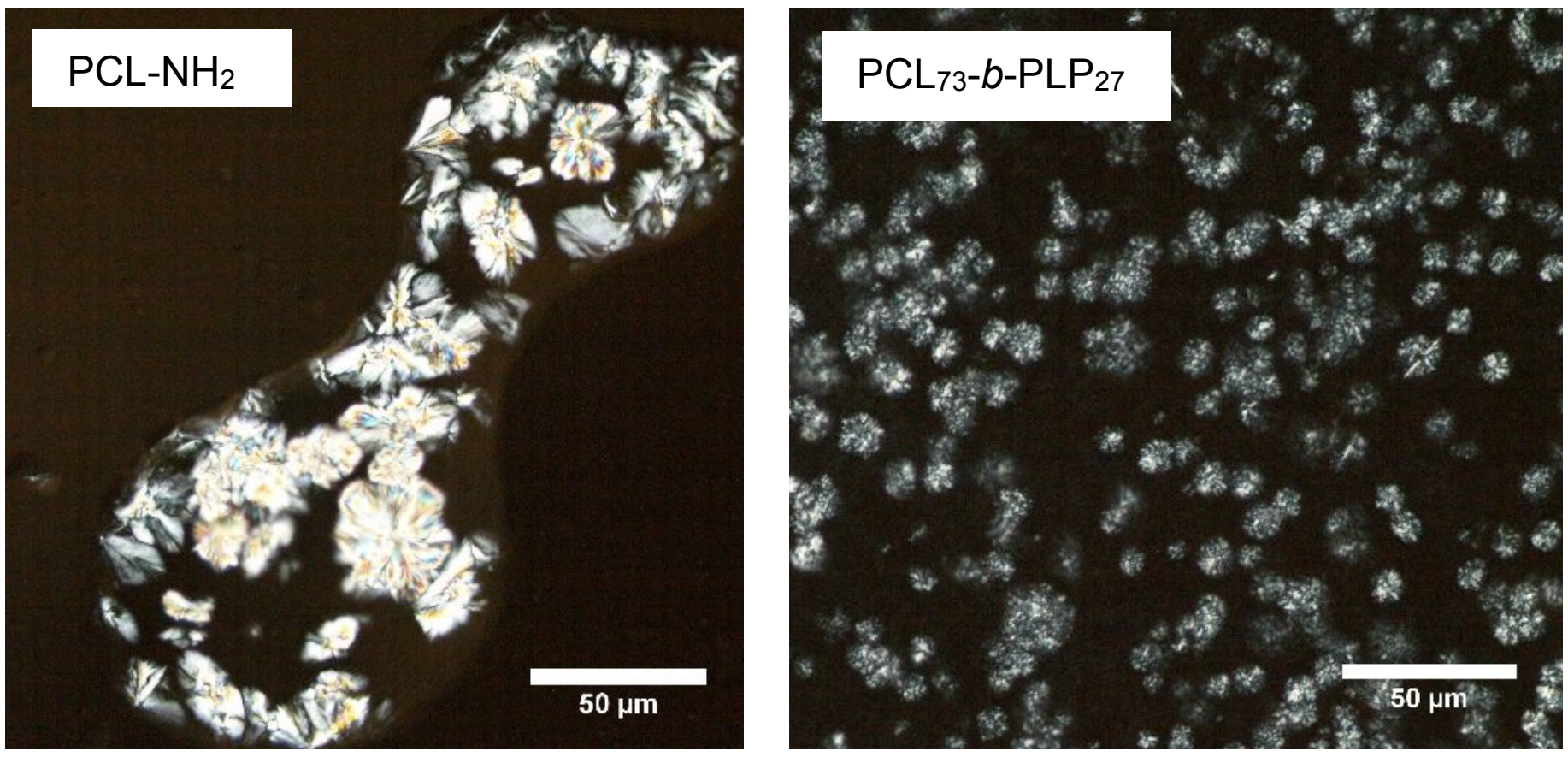

Figure S7. POM micrographs of PCL-NH_2 and PCL_{73}-*b*-PLP_{27} showing the spherulites obtained after isothermal crystallization cooling from the melt at 35 ºC.

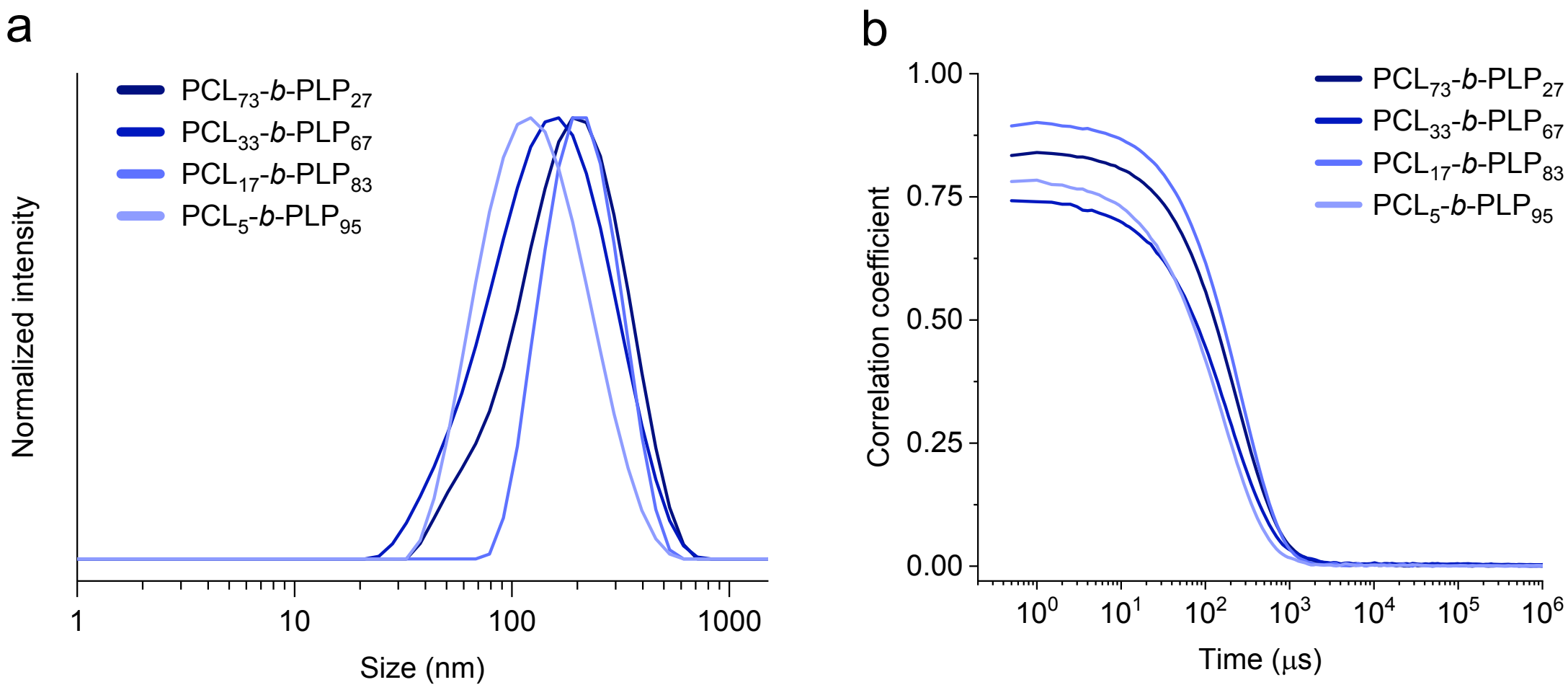

Figure S8. DLS profiles and correlation functions of the PCL_x-*b*-PLP_y copolymers.

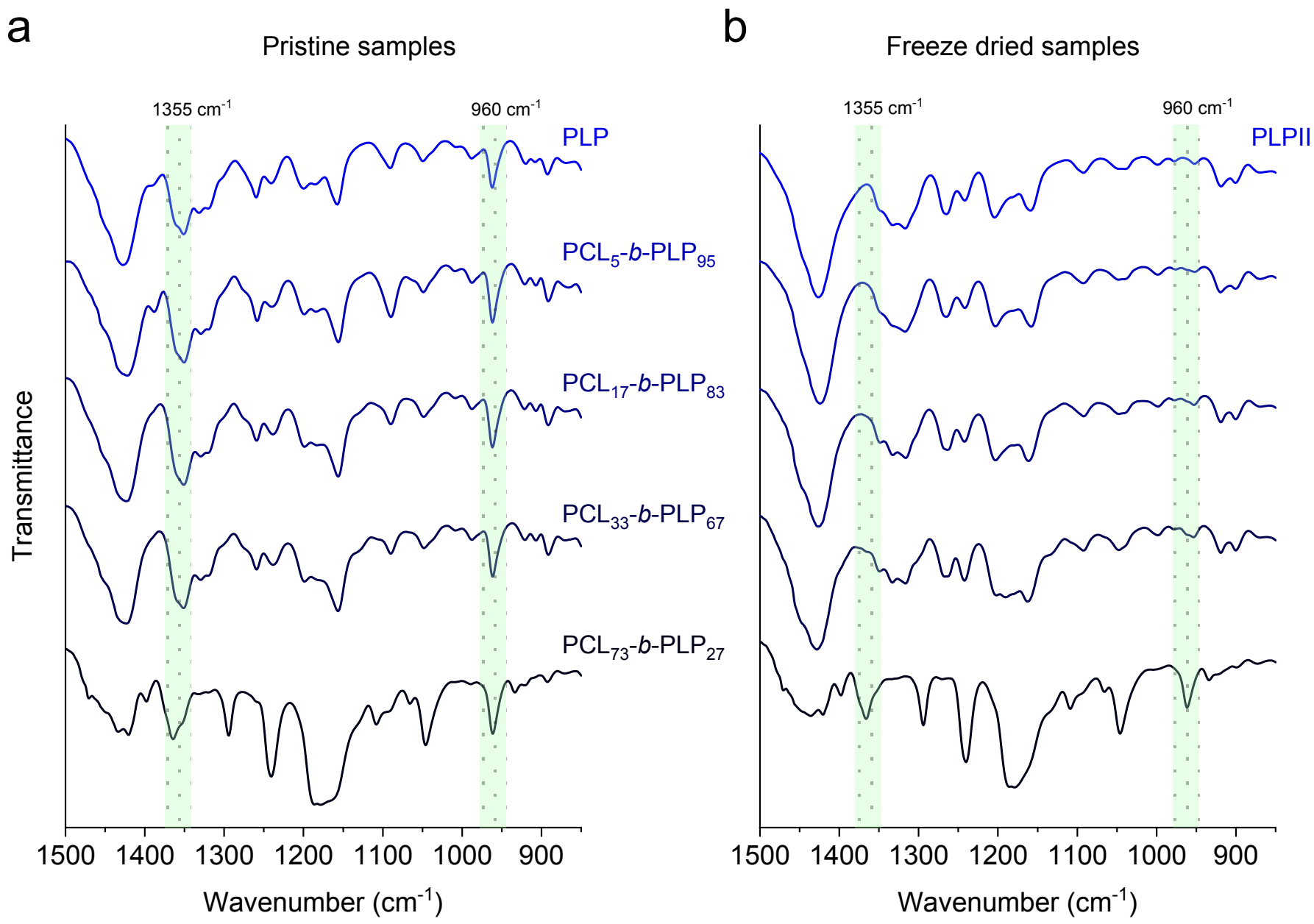

Figure S9. FTIR spectra of: (a) PCL_x-*b*-*cis*-PLP_y and (b) PCL_x-*b*-*trans*-PLP_y copolymers.

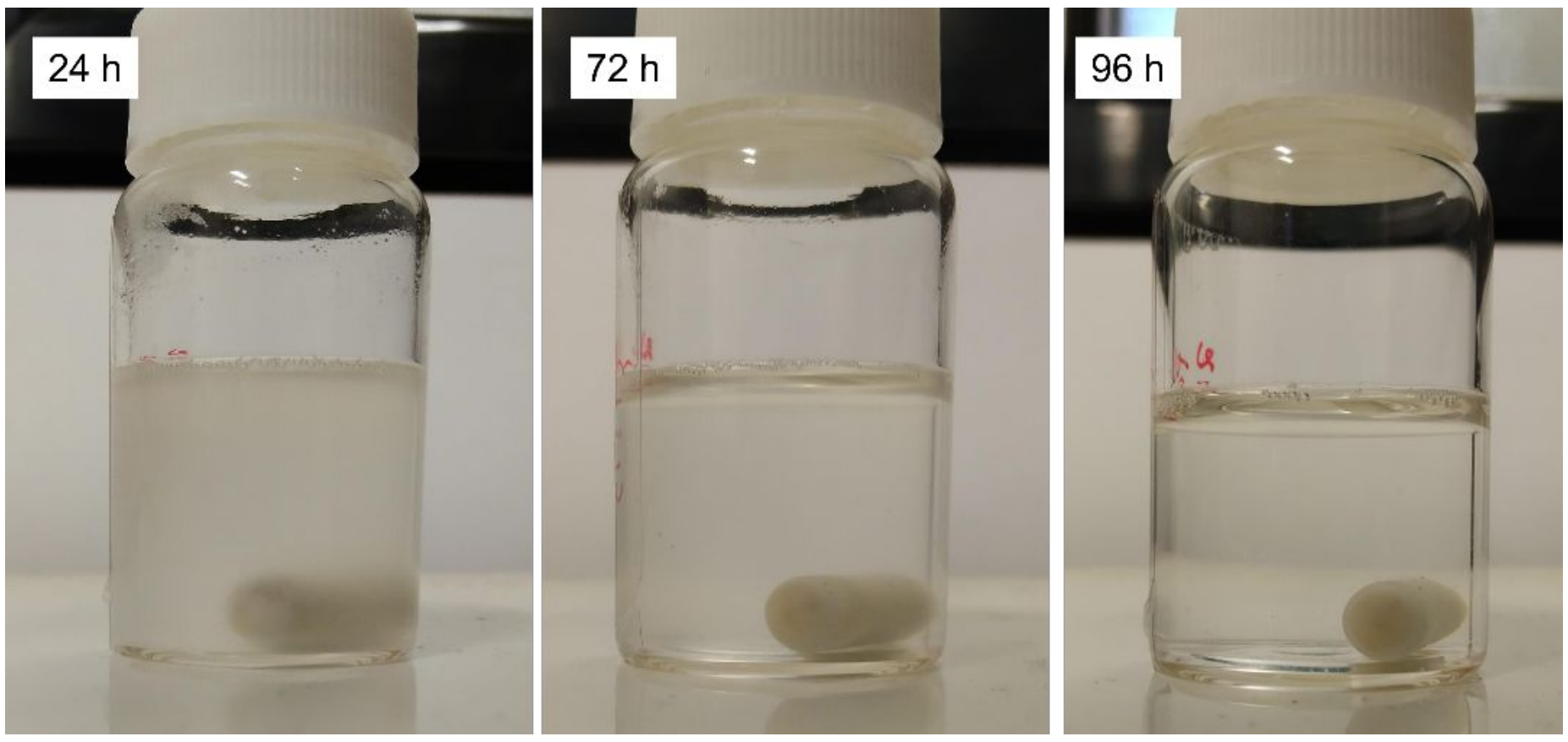

Figure S10. Still photos displaying the effect of the polyproline block mutarotation on the turbidity of the PCL_{33}-*b*-PLP_{67} solution in water.

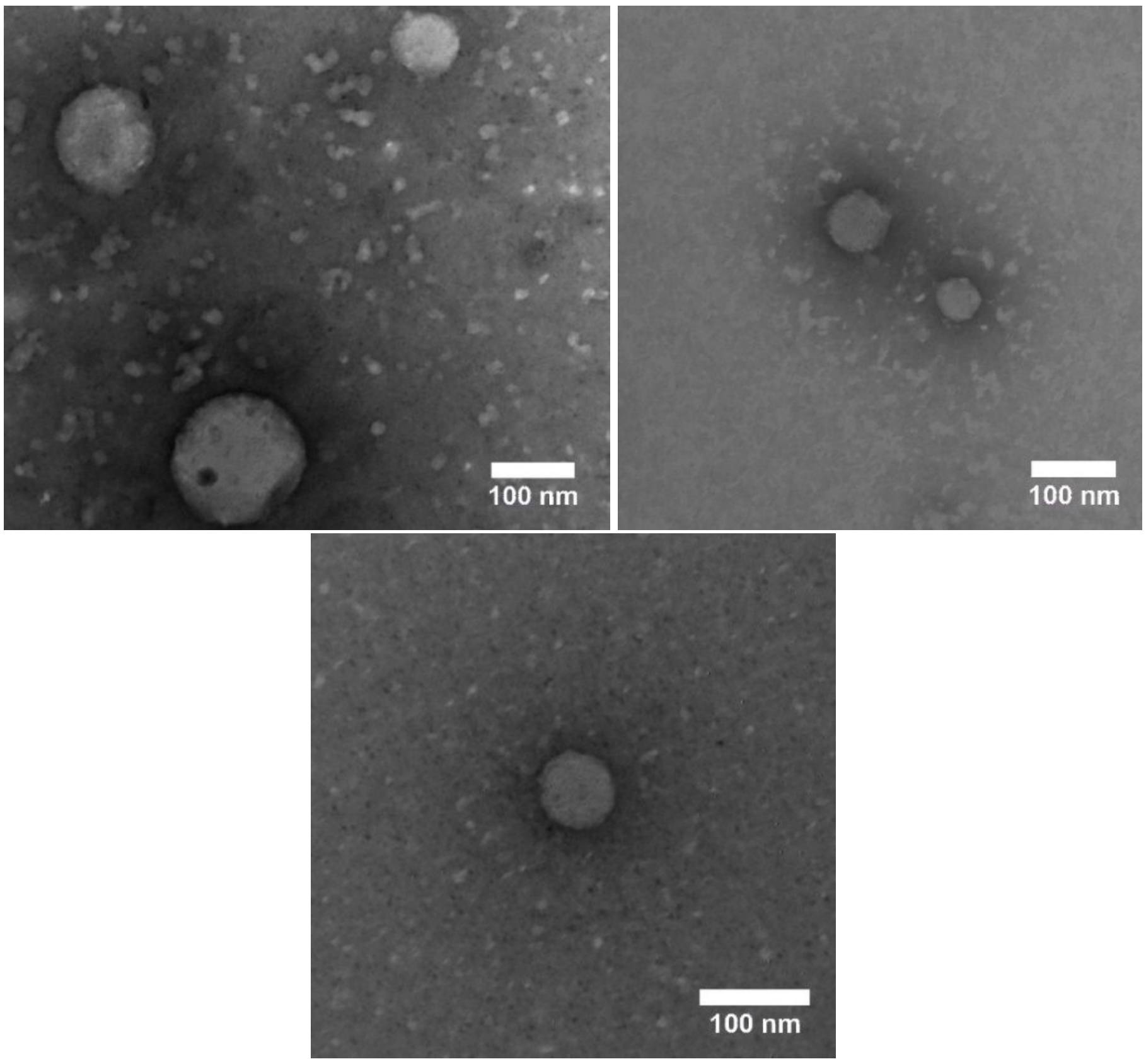

Figure S11. A selection of TEM images of nanoparticles from the PCL_{17}-*b*-PLP_{83} copolymer. Scale bar = 100 nm.

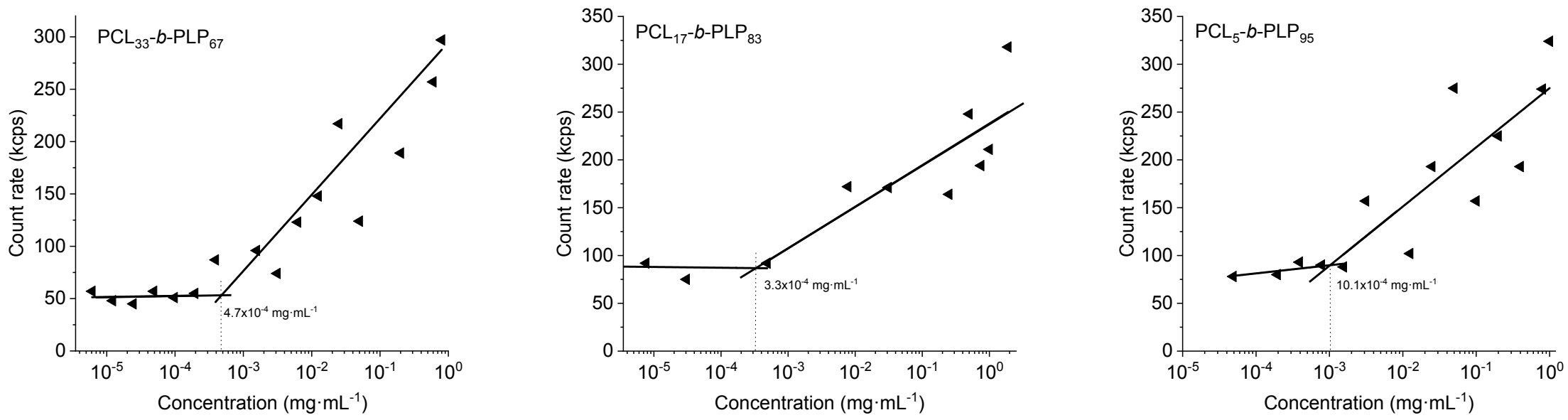

Figure S12. Critical micelle concentration of selected polyproline-based copolymers.

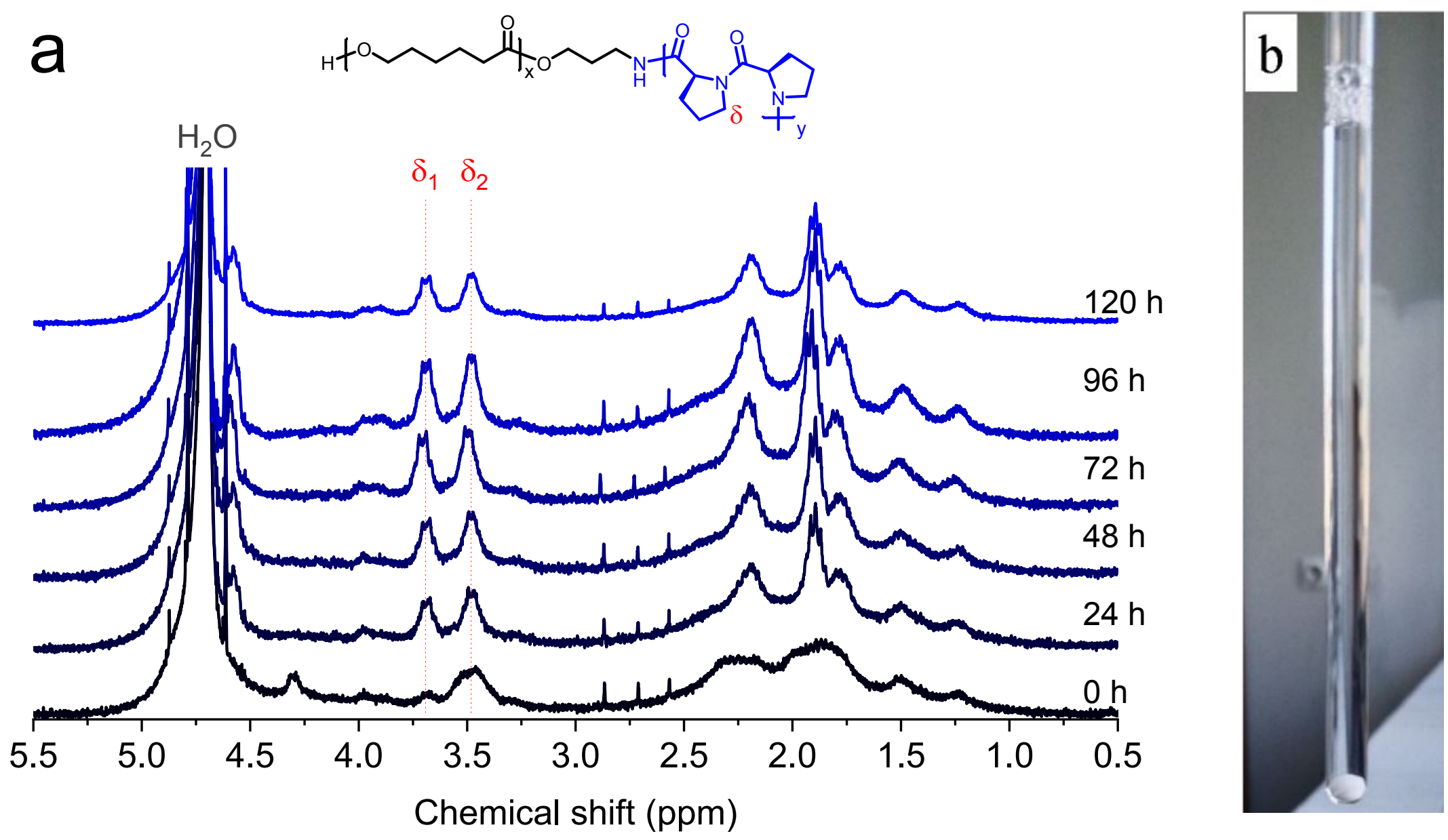

Figure S13. (a) ^{1}H NMR spectra (D_2O, 300 MHz) of the PCL_5-*b*-PLP_{95} diblock copolymer recorded at different time points. (b) Still photo of the NMR tube exhibiting the fully soluble copolymer after 96 h.

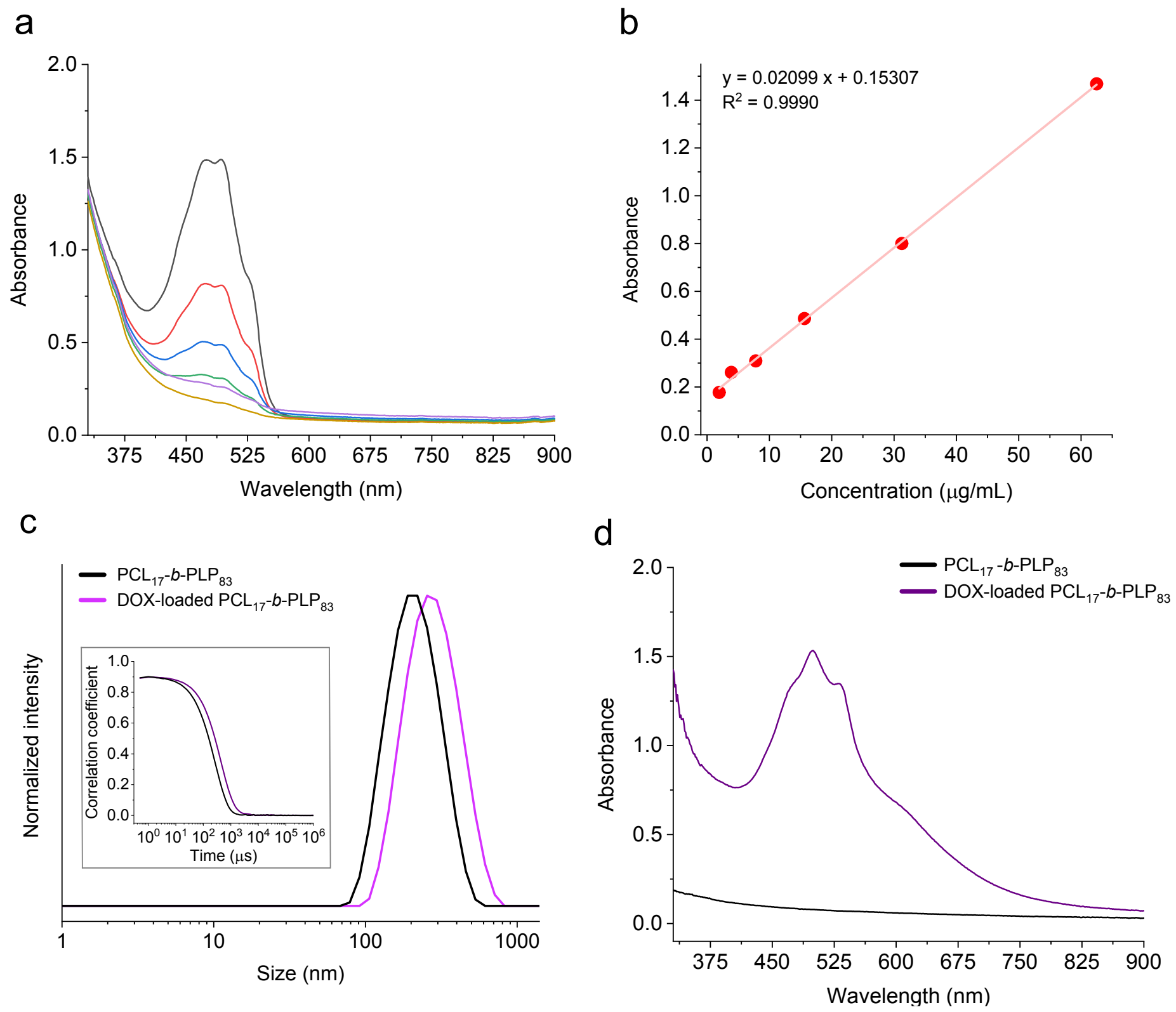

Figure S14. Drug encapsulation in polyproline–based nanoparticles. (a, b) UV-vis spectra of DOX·HCl standards in TFE and the corresponding calibration curve. (c, d) DLS profiles (inset: correlation coefficient) and UV-vis spectra of blank and loaded nanoparticles prepared from the PCL_{17}-*b*-PLP_{83} copolymer.

4. References

(1) Le Hellaye, M.; Fortin, N.; Guilloteau, J.; Soum, A.; Lecommandoux, S.; Guillaume, S. M. Biodegradable Polycarbonate-b-Polypeptide and Polyester-b-Polypeptide Block Copolymers: Synthesis and Nanoparticle Formation Towards Biomaterials. *Biomacromolecules* **2008**, *9* (7), 1924–1933. https://doi.org/10.1021/bm8001792.
